# Genetic testing in women with early-onset breast cancer: a Traceback pilot study

**DOI:** 10.1007/s10549-021-06351-z

**Published:** 2021-09-16

**Authors:** Annelie Augustinsson, Martin P. Nilsson, Carolina Ellberg, Ulf Kristoffersson, Håkan Olsson, Hans Ehrencrona

**Affiliations:** 1 Cancer Epidemiology, Department of Clinical Sciences, Lund University, Lund, Sweden; 2 Care in High Tech Environments, Department of Health Sciences, Lund University, SE-221 00 Lund, Sweden; 3 Oncology and Pathology, Department of Clinical Sciences, Lund University, Lund, Sweden; 4 Hematology, Oncology and Radiation Physics, Skåne University Hospital, Lund, Sweden; 5 Clinical Genetics, Department of Laboratory Medicine, Lund University, Lund, Sweden; 6 Clinical Genetics and Pathology, Laboratory Medicine, Skåne University Hospital, Lund, Sweden

**Keywords:** Breast cancer, Early-onset, Genetic testing, *BRCA1*, *BRCA2*

## Abstract

**Purpose:**

In Sweden, a Traceback approach, i.e., a retrospective genetic outreach activity, among cancer patients is not normally used in clinical practice. In this pilot study, we wanted to evaluate a Traceback strategy for possible future clinical implementation and investigate why not all women with early-onset breast cancer underwent genetic testing when they were first diagnosed.

**Methods:**

Out of all women (*n* = 409) diagnosed with breast cancer at ≤ 35 years in Southern Sweden between 2000 and 2017, 63 had not previously been tested. These women were offered an analysis of the genes *BRCA1*, *BRCA2*, *PALB2*, *CHEK2*, and *ATM* through a standardized letter. Subsequently, women with normal test results were informed through a letter and carriers of pathogenic variants were contacted through a telephone call and offered in-person genetic counseling. All tested women were asked to complete a follow-up questionnaire regarding previously not having attended genetic counseling and testing and their experiences of the current retrospective approach.

**Results:**

Out of the invited women, 29 (46%) underwent genetic testing and 27 (43%) answered the questionnaire. Pathogenic variants were identified in *BRCA1* (*n* = 2), *CHEK*2 (*n* = 1), and *ATM* (*n* = 1). The main reason for previously not having undergone genetic testing was not having received any information from their physicians. Most study participants were satisfied with both written pre- and post-test information.

**Conclusion:**

The process with retrospective identification, written pre-test information, and genetic testing, followed by in-person counseling for carriers of pathogenic variants only, was well accepted. This has implications for future Traceback implementation programs.

**Supplementary Information:**

The online version contains supplementary material available at 10.1007/s10549-021-06351-z.

## Introduction

Although breast cancer is the most common cancer in women, both worldwide and in Sweden, only 1.5% were diagnosed before the age of 35 years in Sweden in 2019 [1]. Unfortunately, breast cancers in young women are often aggressive and associated with a relatively poor prognosis [2–4]. Early-onset breast cancers are also more often associated with heredity, and young patients are more likely to harbor a genetic predisposition to breast cancer [5]. Of all breast cancer cases, 5–10% have a strong hereditary background and a recent study has estimated the prevalence of pathogenic variants in *BRCA1* and *BRCA2* among unselected breast cancer patients to be about 4.5% [6]. The corresponding percentage in patients diagnosed before 35 years is 10–15% [7]. Among women previously diagnosed with breast cancer, the finding of a pathogenic variant is associated with an increased risk for the development of new primary cancers. Therefore, the identification of pathogenic variants in early-onset breast cancer patients is of great importance [5, 7].

For many years, Swedish national breast cancer guidelines have recommended that all women diagnosed with invasive breast cancer at 35 years or younger should be referred for genetic counseling, and given the option of genetic testing, regardless of family history of cancer [8]. These recommendations were widened in 2017, to include all women diagnosed with breast cancer at 40 years or younger [9]. A knowledge of carrier status in previously diagnosed breast cancer patients is highly important for prevention strategies for the development of new primary cancers. For carriers of pathogenic variants, increased surveillance through annual mammography and magnetic resonance imaging (MRI) screening is usually recommended. In addition, these carriers can also be given the option of risk-reducing measures to improve both breast cancer specific and overall survival, i.e., prophylactic contralateral mastectomy and bilateral salpingo-oophorectomy. The identification of a pathogenic variant in *BRCA1*, *BRCA2*, or another gene associated with breast cancer, is also associated with potential benefits for the woman’s family, as it may influence relatives to opt for genetic counseling and testing to identify healthy carriers at high risk, and thereby be able to prevent cancer and cancer-related deaths through increased surveillance and prophylactic surgery [10, 11].

In 2016, a workshop was convened at the US National Cancer Institute to discuss a framework designated ‘Traceback’ for retrospective identification of *BRCA1* and *BRCA2* germline pathogenic variants in previously diagnosed but unreferred ovarian cancer patients and their relatives [12].

This type of retrospective genetic outreach activity in cancer patients is not normally used in clinical practice in Sweden. However, based on our previous experiences [13, 14], we decided to initiate a Traceback pilot study by inviting women diagnosed with breast cancer at 35 years or younger who had not previously been referred for genetic counseling.

The main objective for the study was to evaluate a Traceback counseling strategy, with possible adaptations for broader Traceback studies and future clinical implementation. The secondary objective was to gain a deeper understanding why not all women with early-onset breast cancer attended genetic counseling and testing when they were first diagnosed.

## Materials and Methods

### Data acquisition

National civic registration numbers and information regarding names and treating physicians for all women diagnosed with breast cancer at 35 years or younger in the South Swedish Health Care Region were retrieved from the Southern Swedish Regional Tumor Registry in Lund and from the National Quality Registry for Breast Cancer (NKBC) in Stockholm. Information regarding which patients were registered at the Oncogenetic Clinic in Lund and which were not was subsequently retrieved from the OnkGen Register and from clinical records at Skåne University Hospital in Lund. Vital status, current addresses, and telephone numbers to all the women who were not registered at the clinic were extracted from the Population Register, administered by the Swedish Tax Agency.

### Study cohort

**Fig. 1:**
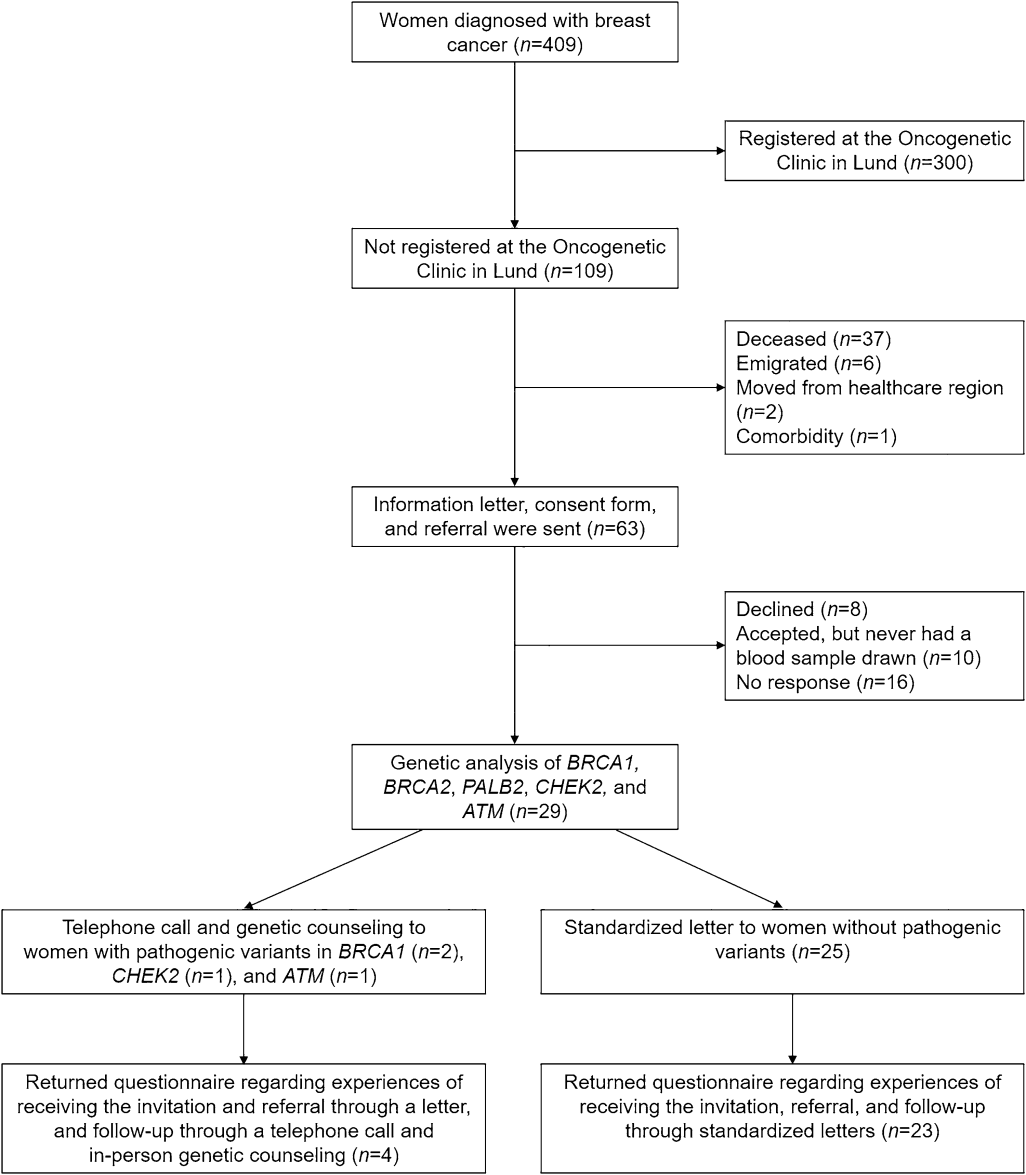
Flowchart summarizing the study cohort, inclusion, genetic analyses, and return of questionnaires

In total, 409 women were diagnosed with invasive breast cancer at 35 years or younger in the South Swedish Health Care Region between January 1, 2000, and December 31, 2017. After exclusion of women who were already registered at the Oncogenetic Clinic at Skåne University Hospital in Lund, deceased, emigrated, or had moved to another healthcare region in Sweden, inquiries were sent to the remaining 64 women’s treating physicians to ensure that no existing medical condition would prevent possible study inclusion. After exclusion of one ineligible patient due to comorbidity, an invitation letter with informed consent was subsequently sent to 63 eligible women (Fig. 1).

### Summary of the Traceback pilot study procedure

An invitation letter (Online Resource 1) was sent by regular mail. The letter contained information about the study and possible implications of genetic testing, an informed consent form, a pre-paid envelope, contact information, and a referral form for a blood sample for DNA extraction. The women were invited to contact the study coordinators for additional information if needed.

If the consent form was returned and a blood sample was drawn, with no cost for the participant, the breast cancer susceptibility genes *BRCA1*, *BRCA2*, *PALB2*, *CHEK2*, and *ATM* were analyzed (for technical details, see Online Resource 2). According to current clinical routine in Sweden [15], genetic testing of the genes *BRCA1*, *BRCA2*, *TP53*, *PALB2*, *CHEK2*, and *ATM* is offered to women with suspected hereditary breast cancer after in-depth genetic counseling. For a more streamlined approach suitable for a Traceback scenario, it was decided by the study management to exclude *TP53* due to the specific difficulties involved with discussing cancer risks and management for carriers of pathogenic variants in this gene (for a more detailed discussion regarding the decision to exclude *TP53*, see Online Resource 2).

After approximately one month, a follow-up telephone call was made to the women who had not returned the consent form.

After one additional month, a reminder with a new written informed consent form, pre-paid envelope, and referral for DNA extraction was sent to each of the women who had not returned the consent form.

Women with normal test results (non-carriers of pathogenic variants) were informed about the test result through a standardized letter (Online Resource 3). To resemble clinical practice as much as possible, the standardized letter used the same phrasing as in clinical templates used by genetic counselors at the Oncogenetic Clinic in Lund, where applicable. This letter also contained a questionnaire with questions regarding the reason for not attending genetic counseling and testing when they were first diagnosed with breast cancer and their attitudes toward the current retrospective approach. Carriers of pathogenic variants were informed about the result through a telephone call from a genetic counselor and given time for an appointment at the Oncogenetic Clinic in Lund at their earliest convenience. These women were handed the questionnaire after the subsequent genetic counseling session.

### Statistical analysis

Descriptive statistics were used to summarize pertinent information from one closed-ended questionnaire question and six scaled-response questions with Likert rating scales ranging from 1 (strongly disagree) to 5 (strongly agree). Spontaneous answers to three open-ended questions were grouped into categories using content analysis. Mann–Whitney *U* test was used to examine the differences in median age at breast cancer diagnosis, median age at study invitation, and median time between breast cancer diagnosis and study invitation between women who completed all parts of the study and those who did not. Fisher’s exact test was used to examine the association between place of residence, i.e., in which county in the South Swedish Health Care Region the women lived, and completing all parts of the study. All analyses were conducted using the IBM SPSS statistical computing package (version 25.0; SPSS, Inc., Chicago, Illinois, USA). Statistical significance was considered with a two-tailed *p*-value of < 0.05.

## Results

### Characteristics of study population

A total of 63 women diagnosed with invasive breast cancer at 35 years or younger in the South Swedish Health Care Region between January 1, 2000 and December 31, 2017, who had not previously been registered at the Oncogenetic Clinic at Skåne University Hospital in Lund, were offered analysis of the genes *BRCA1*, *BRCA2*, *PALB2*, *CHEK2*, and *ATM*. Median ages at diagnosis and study invitation was 34.2 (range: 25.3–35.9) and 45.8 (33.0–54.2) years, respectively. Median time from breast cancer diagnosis to study invitation was 12.3 (2.8–20.3) years.

### Consent for study participation

Detailed information regarding study enrollment, follow-up, genetic analyses, and completion of questionnaires is shown in Online Resource 4. Twenty-six (41%) women returned the signed consent form within a month. Out of these, 24 (92%) accepted participation, whereas two women signed the form but declared that they did not wish to participate in the study. After approximately one month, follow-up telephone calls were made to the women who had not returned the signed consent form. Contacts were made with 14 (38%), of whom five (36%) declined participation. Reasons for declining were a lack of time, the inability to emotionally handle a negative consequence of the test outcome, and not having any daughters. Approximately one month after the follow-up telephone call, written reminders were sent to the women who still had not returned the signed consent form (*n* = 26). This resulted in an additional seven women returning the consent form, of whom six accepted study participations and one declined. Summing up, out of 35 consenting women, 24 (69%) consented without a reminder, five (14%) after the follow-up telephone call, and six (17%) after the written reminder.

### Uptake of genetic testing

Twenty-nine (83%) of the consenting women subsequently had a blood sample drawn for DNA extraction and genetic analysis. Germline pathogenic variants were identified in four (14%); two in *BRCA1*, one in *CHEK2*, and one in *ATM* (for further information, see Online Resource 2). Subsequently, all four women with pathogenic variants and 23 (92%) women without pathogenic variants completed the follow-up questionnaire (Fig. 1, Online Resource 4).

### Characteristics of women who completed the study vs. those who did not

Out of the 27 women who completed all parts of the Traceback study, i.e., accepted participation, underwent genetic testing, and answered the questionnaire, 22 (81%) returned the signed consent form within one month, one (4%) after the follow-up telephone call, and four (15%) after the written reminder (Online Resource 4). There were no statistically significant differences between median ages at breast cancer diagnosis or study invitation of women who completed all parts of the Traceback study compared with those who did not (34.2 *vs*. 34.0; *p* = 0.92, and 46.3 *vs*. 45.7 years; *p* = 0.80, respectively). Neither was there any statistically significant difference between median time from breast cancer diagnosis to study invitation for these women (12.3 *vs*. 12.0 years; *p* = 0.75). In addition, when analyzing the association between completing all parts of the Traceback pilot study or not and place of residence, i.e., in which county they lived, no statistically significant difference was found.

### Reasons for not having been tested previously

The most reported reason (*n* = 20; 74%) for not having been tested when first diagnosed with breast cancer was not receiving any information regarding genetic counseling and testing from treating physicians. One woman (4%) reported that she had received information but declined. Five (20%) reported other reasons for previously not having been referred to the Oncogenetic Clinic in Lund. Two of these reasons were that “They (the physicians) didn’t think it was an enhanced risk that mine was hereditary” and that “They (the physicians) didn’t think it was genetic, since no one in the family has had cancer”. Two other women reported that they did not remember if they discussed a referral with their physicians. One woman wrote “I have gone through breast cancer operations three times. I don’t have any breasts left. I’m scared of developing tumors somewhere else in the body”, which might not be an answer to the question per se. One woman did not report any reason at all for not being referred to the clinic.

### Satisfaction with the Traceback approach

Study participants’ answers to the scaled-response questions are listed in Table 1, where the most frequent answers are highlighted in bold. Most women, both with and without germline pathogenic variants, reported that they understood and were satisfied with the written study information, as well as the opportunity for additional contacts and going through with the genetic testing. However, six (26%) of the women without a pathogenic variant agreed to wanting additional oral information. Most women also reported that they had shared the information with their relatives and that they would recommend a female friend with breast cancer to undergo genetic testing in the same way that they did (Table 1).

**Table 1:** Closed-ended scaled-response questions with answers ranging from 1 (strongly disagree) to 5 (strongly agree)

Statements	1	2	3	4	5
Women without a pathogenic variant, *n* = 23					
I understood the written information that I obtained regarding the study				5 (21.7%)	**18 (78.3%)**
I am satisfied with the obtained written information regarding the study and the opportunity for further contacts				3 (13.0%)	**20 (87.0%)**
I would have wished for additional oral information	**13 (56.5%)**	4 (17.4%)	5 (21.7%)	1 (4.3%)	
I am satisfied with undergoing genetic testing				1 (4.3%)	**22 (95.7%)**
I have shared the information that I obtained with my relatives	2 (8.7%)	2 (8.7%)		2 (8.7%)	**17 (73.9%)**
I would recommend a female friend with breast cancer to undergo genetic testing in the same way that I did			3 (13.0%)	2 (8.7%)	**18 (78.3%)**
Women with a pathogenic variant, *n* = 4					
I understood the written information that I obtained regarding the study				1 (25.0%)	**3 (75.0%)**
I am satisfied with the obtained written information regarding the study and the opportunity for further contacts					**4 (100.0%)**
I would have wished for additional oral information	**4 (100.0%)**				
I am satisfied with undergoing genetic testing					**4 (100.0%)**
I have shared the information that I obtained with my relatives				1 (25.0%)	**3 (75.0%)**
I would recommend a female friend with breast cancer to undergo genetic testing in the same way that I did				1 (25.0%)	**3 (75.0%)**

Most frequent answers are highlighted in bold

Open-ended questions and selected spontaneous answers from the study participants are listed in Table 2. Regarding the women’s experiences of being offered genetic testing through a letter, the answers were grouped into the categories “Positive responses” (*n* = 23, 85%) and “Emotional but positive responses” (*n* = 4, 15%). The main reasons why the women chose to participate in the Traceback pilot study and undergo genetic testing were that they saw either a benefit for their family members or themselves, or that they saw a need for more knowledge. These reasons were categorized as “Family’s risk”, “Own and family’s risk”, “Own risk”, and “Increased knowledge”, respectively (Table 2). Unselected spontaneous answers are listed in Online Resource 5. One woman wrote an additional comment at the end of the questionnaire; that she would have wanted to get the offer of genetic testing while undergoing her treatment, not after several years.

**Table 2:** Open-ended questions and selected spontaneous answers from the study participants

Questions	Categories	Illustrative quotes
Women without a pathogenic variant, *n* = 23		
What was your experience of being offered genetic testing through a letter?	Positive responses (*n* = 20)	Super happy, since I have a daughter and was afraid for her sake Fantastic! I felt a huge reverence over this offer, considering it was a long time ago since I got my cancer diagnosis and went through all the treatments – THANK YOU! Wonderful! Really good to know
	Emotional but positive responses (*n* = 3)	At first, I was afraid, didn’t want to “wake” everything to life again. But, after we talked about it at home, it was a given
What was the main reason for choosing to participate in the study?	Family’s risk (*n* = 7)	I have a daughter (who was 9 months old when I realized that I had a lump in my breast), whom I obviously have been worried for, considering the heredity of breast cancer Because of my daughter and my sister’s daughters
	Own and family’s risk (*n* = 5)	Of course, you want to know if you have an enhanced risk for additional cancer diagnoses, or if my children have
	Own risk (*n* = 2)	Foremost, to know if I should do any preventive efforts to avoid being affected again. Operations on the other breast or uterus
	Increased knowledge (*n* = 7)	Knowledge is always positive I’m adopted. Didn’t know if I had a hereditary alteration in the genes
	Support research (*n* = 2)	To support medical research. I’m going through investigation due to metastasis in the lung, so I’m already enrolled within the health care system
What was your experience of being informed of the result from the genetic analysis through a letter?	Positive responses (*n* = 20)	Good. Got the information before, on how I was going to be contacted if I did/if I didn’t carry the gene A relieve that it wasn’t genetic
	Additional oral conversation (*n* = 2)	OK, since it was a positive answer. But, a conversation is preferred
	Emotional but positive response (*n* = 1)	It obviously felt a bit jittery and worrisome, but at the same time it was nice that the information for my part was calming
Women with a pathogenic variant, *n* = 4		
What was your experience of being offered genetic testing through a letter?	Positive responses (*n* = 3)	I was happy that I got to see if it was genetic Positive
	Emotional but positive response (*n* = 1)	Sad to tear up old worries, but grateful that it’s checked out, not least for my daughter’s sake
What was the main reason for choosing to participate in the study?	Family’s risk (*n* = 2)	To know if you have the gene could protect sisters and daughter in the future
	Own and family’s risk (*n* = 1)	To get knowledge and be able to prevent the preventable, and inform the children so that they are attentive to symptoms
	Own risk (*n* = 1)	I want to prevent the risk for additional breast cancer and ovarian cancer
What was your experience of being informed of the result from the genetic analysis through a telephone call and subsequent genetic counseling?	Positive responses (*n* = 2)	Good information It was good
	Emotional but positive response (*n* = 1)	More than a week between the first conversation with the nurse and the conversation with the physician. Had time to think and worry a little during this time. Otherwise, totally OK
	Emotional response (*n* = 1)	I was a bit sad

The 23 women with normal genetic test results answered a question regarding their experiences of being informed of the result through a standardized letter. All these women had a positive component to their answer, but two (9%) were categorized as “Additional oral conversation” and one (4%) as “Emotional but positive response”. The four women who were tested with pathogenic variants instead answered a question regarding their experiences of being informed of the result through a telephone call from a genetic counselor and the subsequent in-person genetic counseling. These answers were categorized as “Positive responses” (*n* = 2), “Emotional but positive response” (*n* = 1), and “Emotional response” (*n* = 1) (Table 2, Online Resource 5).

## Discussion

In the present Traceback pilot study, we invited all women diagnosed with breast cancer at 35 years or younger in the South Swedish Health Care Region to undergo genetic testing, if they were not previously registered at the Oncogenetic Clinic at Skåne University Hospital in Lund. We also evaluated why they had not been registered at the clinic, even though they fulfilled the Swedish national guidelines for consideration of genetic counseling and testing, and their attitudes toward the current retrospective approach.

Out of the 63 invited women, 27 (43%) completed all parts of the Traceback pilot study. There was no significant difference between median age at study invitation for the women who completed all parts of the study compared with those who did not. In previous studies regarding rapid genetic testing of newly diagnosed breast cancer patients, it has been reported that women who accepted study participation were younger than those who declined [16, 17]. The discrepancy between our study and these previous studies could potentially be attributed to the fact that all women in our study were relatively young (33.0–54.2 years).

In our study, 29 (46%) women underwent genetic testing. When comparing this result with a previous Swedish study regarding *BRCA1/2* testing after written pre-test information without face-to-face counseling (the BRCAsearch study), the uptake seems remarkably low [16, 18]. It should be noted, however, that the BRCAsearch study concerned newly diagnosed women and our study used a retrospective approach. Since none of the invited women chose to answer the questionnaire without completing the genetic testing, we do not know why these women declined participation. Previous studies have reported several factors as associated with patients declining genetic counseling, including patients’ socioeconomic status [19], the expected benefits or limitations of genetic counseling and testing, a fear of psychological effects, a lack of time, and a limited ability to travel [20, 21]. Consistent with some of these studies, the women who declined participation at the follow-up telephone call in our study (*n* = 5) stated a lack of time, the inability to emotionally handle a negative consequence of the test outcome, and not having any daughters. Additionally, even though most of the women stated that they did not get any information about genetic counseling and testing when they were first diagnosed, we cannot rule out the possibility that some women who chose not to participate may have been offered testing previously but declined. Finally, the COVID-19 pandemic came as an unexpected event during the study inclusion period. It is possible that the pandemic affected the study outcome, so that fewer women accepted participation compared with what would have been the case otherwise.

Even though the uptake for genetic testing was relatively low, it increased from 22 (35%) to 24 (38%) women after the follow-up telephone call, and to 29 (46%) women after the written reminder. Considering this, it may be sufficient with only one written reminder in future Traceback implementations to simplify the procedure.

On average, 10–15% of women diagnosed with breast cancer before 35 years are carriers of pathogenic variants in *BRCA1* or *BRCA2* [5]. Four (14%) of the women who underwent genetic testing in our study were identified as carriers of pathogenic variants. Out of these, two (7%) were carriers of pathogenic variants in *BRCA1* and none in *BRCA2*, a figure that might seem low. Since the study sample is small, it is possible that this is simply due to chance. However, it is also possible that the women who were not offered genetic counseling when they were first diagnosed more often lacked a family history suggesting hereditary breast and ovarian cancer, which could explain a lower genetic diagnostic yield. This hypothesis is supported by comments made by two of the participants in the questionnaire, i.e., “They (the physicians) didn’t think it was hereditary/genetic”.

Consistent with the findings in our study, a previous study regarding simplified pre-test information and germline *BRCA1/2* testing reported that very few patients contacted them for genetic counseling or with practical questions over the telephone, suggesting that the majority felt that the written pre-test information was sufficient [18]. The main reasons why the women chose to participate in the study and undergo genetic testing were that they saw either a benefit for their family members, especially daughters, or themselves, which is consistent with a previous study [22]. Out of the women who were tested without pathogenic variants, most reported that they were content with being informed through a letter. Out of the four women who were tested with pathogenic variants, two were clearly satisfied with the procedure, where they were informed of the result through a telephone call from a genetic counselor and the subsequent in-person genetic counseling. One woman was concerned about the time between the telephone call and the in-person counseling, and one woman answered that she “was a bit sad”, which might not have reflected the question per se. Considering the above results, it appears that most of the consenting women were satisfied with both the written pre- and post-test information.

A major limitation in this study is that only 27 (43%) of 63 invited women completed the full procedure, including genetic testing and return of questionnaires. Even though no woman expressed any serious concerns regarding her experiences with being contacted retrospectively and receiving written information by regular mail, we cannot exclude the possibility that some of the women who never responded may have reacted differently.

In this pilot study we wanted to evaluate a Traceback counseling strategy by inviting all women diagnosed with breast cancer at 35 years or younger in the South Swedish Health Care Region who were not previously registered at the Oncogenetic Clinic in Lund. Pathogenic variants were identified in four (14%) of the participants, i.e., women who would otherwise not be aware of their carrier status.

In summary, this study demonstrated that a retrospective identification of individuals with breast cancer that may benefit from genetic testing, combined with a simplified genetic counseling procedure based mostly on written information followed by in-person counseling for carriers of pathogenic variants only, was well received by the vast majority of consenting study participants. Especially for carriers of pathogenic variants in *BRCA1* or *BRCA2*, it is well established that knowledge of carriership is of major clinical importance, since increased surveillance and prophylactic surgery leads to significantly reduced morbidity and mortality [5, 7]. After some minor adjustments of the study protocol, our next step will be to initiate a larger Traceback implementation study by inviting all previously untested women diagnosed with breast cancer between 36 and 40 years of age. The Swedish national breast cancer guidelines were recently updated with a recommendation for genetic counseling also for this age group, and we expect that a large percentage of women in this group will never have received an offer regarding genetic testing since they were diagnosed before the guidelines changed, further underscoring the unmet clinical need for similar Traceback approaches.

## Supplementary Information

Below is the link to the electronic supplementary material.

Supplementary file1 (DOCX 32 kb)

Supplementary file2 (DOCX 27 kb)

Supplementary file3 (TIF 459 kb) Flowchart detailing the outcome of the various steps in the Traceback pilot study procedure.

Supplementary file4 (DOCX 32 kb)

## Data Availability

Research data will be made available from the corresponding author upon reasonable request due to privacy/ethical restrictions.
