# Supplementary material for: Genetic testing in women with early-onset breast cancer: a Traceback pilot study
Source: Breast Cancer Res Treat. 2021 Sep 16;190(2):307–15. doi: 10.1007/s10549-021-06351-z (PMC8443966; doi:10.1007/s10549-021-06351-z)
Supplement: Supplementary file 1 — Supplementary file1 (DOCX 32 kb) [file 10549_2021_6351_MOESM1_ESM.docx]

**Online Resource 2: Genetic analysis**

DNA was extracted from peripheral blood and quantified by Qubit (Life Technologies, Carlsbad, CA, USA). 275 ng DNA was mechanically fragmented on a Covaris E220evolution (Covaris, Woburn, MA, USA). KAPA HyperPrep (Roche Sequencing, Pleasanton, CA, USA), followed by a custom Twist (Twist Bioscience, San Francisco, CA, USA) library kit, was used to capture the entire genomic sequence of selected genes coupled to an inherited predisposition to cancer (Lund HereditarySolidCancer v1.0). Eight samples were multiplexed, the library pool was quantified by Qubit and fragment sizes determined by Tapestation (Agilent Technology, Santa Clara, CA, USA). Sequencing was performed on an Illumina NovaSeq 6000 (Illumina, San Diego, CA, USA) with 2 x 150 bp paired-end reads. Bcl conversion and demultiplexing was done with bcl2fastq (Illumina), and sequence reads were aligned to human reference genome build GRCh38 using the Burrows-Wheeler Aligner (<https://sourceforge.net/projects/bio-bwa/>). Duplicated reads were identified using Sentieon LocusCollector/Dedup (Sentieon Inc., San Jose, CA, USA) and excluded from downstream analysis. Base quality scores were recalibrated with Sentieon QualCal. The resulting bam file was used to calculate quality parameters using Sentieon’s QC-modules. Runs were accepted if all the following quality parameters were met:

- Average coverage ≥1000 x
- Percentage bases with >500x coverage ≥98%
- Percent on target ≥45%
- Percent duplicates ≤50%
- Fragment size ≥350 bp

**Variant calling and annotation for SNVs and small indels**

Variant calling for single nucleotide variants (SNVs) and indels was performed with Sentieon DNAscope (Sentieon) according to the suggested workflow. For complex SNV/indels, variant calling was also done with FreeBayes (<https://docs.csc.fi/apps/freebayes/>). Detected variants were annotated and ranked using an in-house developed bioinformatic pipeline including the open source tools VEP (<https://www.ensembl.org/info/docs/tools/vep/index.html>), SnpSift (<https://pcingola.github.io/SnpEff/>), CADD (<https://cadd.gs.washington.edu/>), and Genmod (<https://github.com/moonso/genmod>).

**Variant calling and annotation for CNVs**

Variant calling for copy number variants (CNVs) and other structural variants was performed with three different callers: CNVkit [1], Delly [2], and Manta [3], and calls that overlapped >70% were collated using the in-house software SVDB. In addition, MELT [4] was used to detect transposable elements. Detected variants were annotated and ranked with an in-house bioinformatic pipeline utilizing VEP, AnnotSV [5], Pre-score (a local Perl script), and Genmod.

**Visualization of annotated variants**

The annotated vcf files were presented for clinical interpretation using the open-source software Scout (<https://github.com/Clinical-Genomics/scout>). While the custom Twist panel contains other genes related to inherited predisposition to cancer, for the Traceback study, a gene list was constructed so only variants affecting the genes *BRCA1*, *BRCA2*, *PALB2*, *ATM*, and *CHEK2* were visualized. As mentioned in the main paper, the clinical breast cancer predisposition gene panel in Sweden currently contains these five genes, as well as *TP53*. The decision to exclude *TP53* was not made lightly, but previous experience has told us that the specific genetic counseling issues associated with testing for pathogenic variants in *TP53* makes it difficult to include this gene in a streamlined approach without in-person counseling, as in this Traceback study. Pathogenic variants in *TP53* are associated with Li-Fraumeni syndrome, a hereditary condition that involves very high risks of cancer, including in childhood. In a Swedish unpublished study (SWEA), women with suspected inherited breast cancer that were referred for clinical testing of *BRCA1* and *BRCA2* were invited to get a broader genetic investigation including all these genes, after detailed in-person counseling. Over 95% of the Swedish women accepted to participate in the study. However, out of the few that declined, most did so with reference to *TP53*. In the SWEA study subgroup of women with breast cancer at the age of 35 years or younger, about 18% had a pathogenic variant in *BRCA1, BRCA2, PALB2, ATM*, or *CHEK2*, whereas less than 1.5% had a pathogenic variant in *TP53* (unpublished data, Hans Ehrencrona). In summary, we therefore deemed it more important to maximize inclusion and identify carriers of pathogenic variants in the included genes than to complicate the procedure. Furthermore, we lended support in the fact that in many other countries, *TP53* is excluded from the routine clinical breast cancer predisposition gene panels for this very reason. Finally, in the standard letter sent to women without a pathogenic variant in any of the included genes, we encouraged them to contact the Oncogenetic Clinic in Lund if they had information about additional cancer diagnoses in the family, especially if early-onset.

Based on previous quality controls and clinical experience, all variants with a relevant rank score were examined and assessed for pathogenicity on a scale graded 1-5 using either the ACMG [6] or the ENIGMA guidelines (<http://enigmaconsortium.org/>). The turn-around time for the laboratory analysis, i.e., from registration of the blood sample to the delivery of a clinical report, was 23 days in average (range: 15-34 days).

**Clinically relevant variants detected in this study**

| **Gene (transcript)** | **HGVSc** | **HGVSp** | **Classification** |
| --- | --- | --- | --- |
| *BRCA1* (NM_007294.3) | c.3048_3052dup | p.(Asn1018Metfs*8) | Pathogenic (5) |
| *BRCA1* (NM_007294.3) | c.5251C>T | p.(Arg1751*) | Pathogenic (5) |
| *CHEK2* (NM_007194.3) | c.1100del | p.(Thr367Metfs*15) | Pathogenic (5) |
| *ATM* (NM_000051.3) | c.5932G>T | p.(Glu1978*) | Pathogenic (5) |

**References**

1. Talevich E, Hunter Shain A, Botton T, Bastian BC (2014) CNVkit: Genome-wide copy number detection and visualization from targeted sequencing. PLoS Comput Biol. 12(4):e1004873. <https://doi.org/10.1371/journal.pcbi.1004873>
2. Rausch T, Zichner T, Schlattl A, Stütz AM, Benes V, Korbel JO (2012) DELLY: structural variant discovery by integrated paired-end and split-read analysis. Bioinformatics. 28(18):i333-i339. https://doi.org/10.1093/bioinformatics/bts378
3. Chen X, Schulz-Trieglaff O, Shaw R, Barnes B, Schlesinger F, Källberg M, Cox AJ, Kruglyak S, Saunders CT (2016) Manta: rapid detection of structural variants and indels for germline and cancer sequencing applications. Bioinformatics. 32(8):1220-2. https://doi.org/10.1093/bioinformatics/btv710
4. Gardner EJ, Lam VK, Harris DN, Chuang NT, Scott EC, Pittard WS, Mills RE, 1000 Genomes Project Consortium & Devine SE (2017) The Mobile Element Locator Tool (MELT): Population-scale mobile element discovery and biology. Genome Res. 27(11):1916-1929. https://doi.org/10.1101/gr.218032.116
5. Geoffroy V, Herenger Y, Kress A, Stoetzel C, Piton A, Dollfus H, Muller J (2018) AnnotSV: an integrated tool for structural variations annotation. Bioinformatics. 34(20):3572-3574. https://doi.org/10.1093/bioinformatics/bty304
6. Richards S, Aziz N, Bale S, Bick D, Das S, Gastier-Foster J, Grody WW, Hegde M, Lyon E, Spector E, Voelkerding K, Rehm HL, [ACMG Laboratory Quality Assurance Committee](https://pubmed.ncbi.nlm.nih.gov/?term=ACMG+Laboratory+Quality+Assurance+Committee%5BCorporate+Author%5D) (2015) Standards and guidelines for the interpretation of sequence variants: a joint consensus recommendation of the American College of Medical Genetics and Genomics and the Association for Molecular Pathology. Genet Med. 17(5):405-24. https://doi.org/10.1038/gim.2015.30
