# Supplementary material for: Genetic testing in women with early-onset breast cancer: a Traceback pilot study
Source: Breast Cancer Res Treat. 2021 Sep 16;190(2):307–15. doi: 10.1007/s10549-021-06351-z (PMC8443966; doi:10.1007/s10549-021-06351-z)
Supplement: Supplementary file 2 — Supplementary file2 (DOCX 27 kb) [file 10549_2021_6351_MOESM2_ESM.docx]

**Online Resource 3: Information letter to women with normal test results**

Dear N.N.,

You have been diagnosed with breast cancer before 36 years of age and have now participated in the research study “Why do not all young women with breast cancer receive genetic counseling?”

As a part of this study you were offered genetic analysis, because a relatively large proportion of women with early-onset breast cancer have a hereditary alteration (mutation) which is associated with an increased risk for this disease. Fortunately, we did not find a hereditary alteration in any of these genes (the genes are called *BRCA1, BRCA2, PALB2, CHEK2*, and *ATM*).

The above result should primarily be interpreted as reassuring information for you and for your family. Since we have now excluded the presence of known hereditary high-risk factors, the statistical risk of developing breast cancer for your female relatives is only slightly to moderately increased compared with the risk for any woman in the population. At this level of risk, we encourage them to participate in the national mammography screening program from 40 years of age (if deemed necessary, combined with ultrasound), but we do not have any recommendations for further measures in addition to this.

This assessment is based only on our knowledge that you were diagnosed with breast cancer before 36 years of age. If you know that your relatives have an unusually high number of cancer diagnoses, especially if they were early onset, this could lead to another assessment. You are always welcome to contact the Oncogenetic Clinic if you need a further discussion, or if you have additional questions.

Sincerely,

Annelie Augustinsson, MSc, Doctoral student

Dr. Hans Ehrencrona, MD, PhD, Senior Consultant Clinical Geneticist
