# Supplementary figures and images for: Genetic testing in women with early-onset breast cancer: a Traceback pilot study
Source: Breast Cancer Res Treat. 2021 Sep 16;190(2):307–15. doi: 10.1007/s10549-021-06351-z (PMC8443966; doi:10.1007/s10549-021-06351-z)

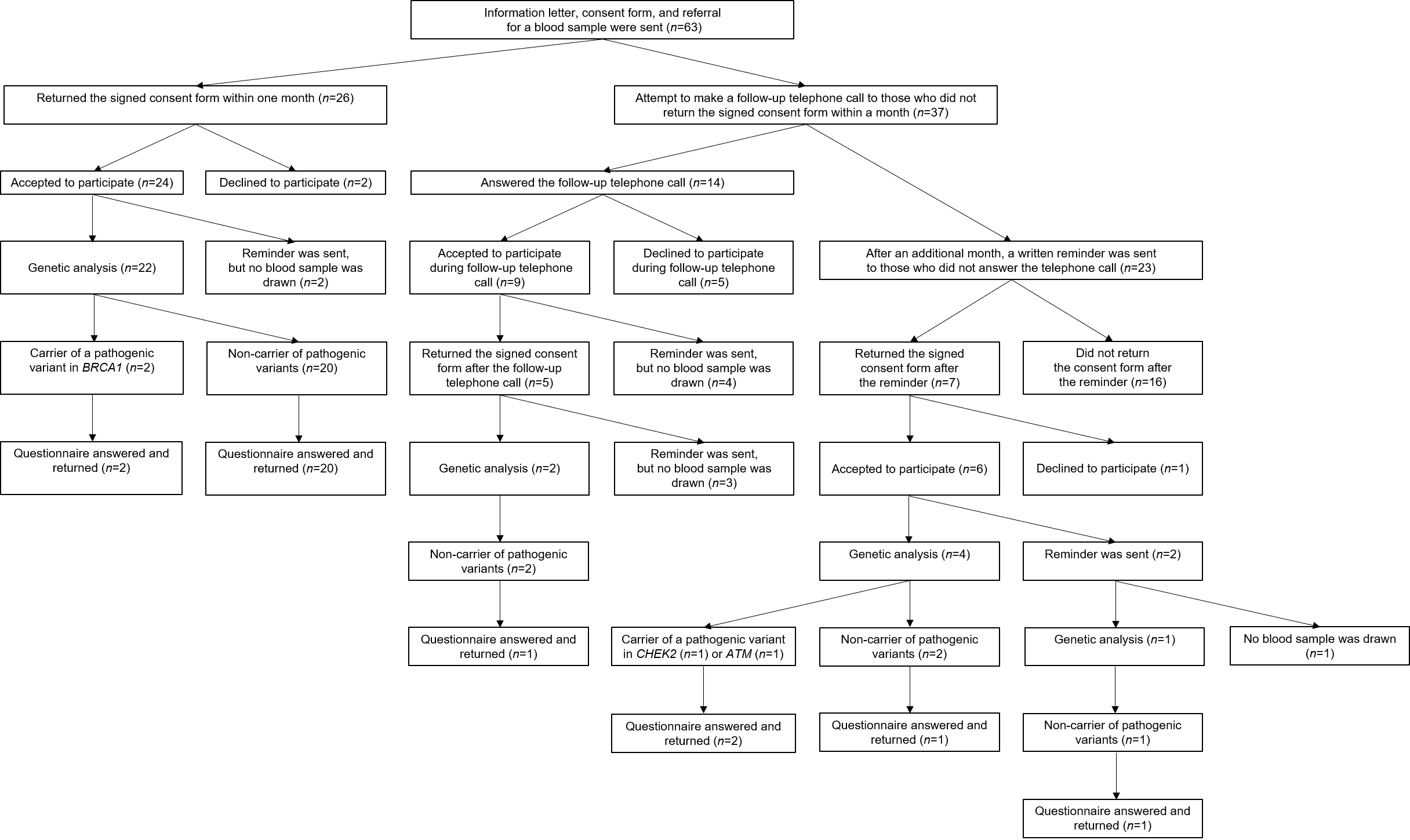

Supplement: Supplementary file 3 — Supplementary file3 (TIF 459 kb) Flowchart detailing the outcome of the various steps in the Traceback pilot study procedure. [file 10549_2021_6351_MOESM3_ESM.tif]
