# Supplementary material for: Genetic testing in women with early-onset breast cancer: a Traceback pilot study
Source: Breast Cancer Res Treat. 2021 Sep 16;190(2):307–15. doi: 10.1007/s10549-021-06351-z (PMC8443966; doi:10.1007/s10549-021-06351-z)
Supplement: Supplementary file 4 — Supplementary file4 (DOCX 32 kb) [file 10549_2021_6351_MOESM4_ESM.docx]

Online Resource 5. Open-ended questions and spontaneous answers from all study participants.

| Answers | Codes | Categories |
| --- | --- | --- |
| **Women without a pathogenic variant, *n*=23** |  |  |
| *What was your experience of being offered genetic testing through a letter?* |  |  |
| Wonderful! Really good to know. | Wonderful and good | Positive responses |
| Good. Never too late for an investigation/testing. | Good |  |
| Good. | Good |  |
| I thought it was good. | Good |  |
| It felt good to check it out. | Good |  |
| Good, since I just had my 5-year check-up and the breast clinic is ending it. | Good |  |
| Good. I have considered my daughter’s risk, and my mom’s. The offer was good, I would not have had the strength to take care of it on my own. | Good |  |
| Thought it was good, considering family and the rest of the world. | Good |  |
| Was happy to be able to do the test. | Happy |  |
| Super happy, since I have a daughter and was afraid for her sake. | Happy |  |
| Very positive. | Positive |  |
| Only positive. | Positive |  |
| Positive. | Positive |  |
| Positive for me and my daughter. | Positive |  |
| Very positive. Have always been a bit worried of transferring the disease to my children. | Positive |  |
| Positive. It feels safe to get it checked out for the rest of the family and for my own sake. | Positive and safe |  |
| I had a positive attitude. A bit shocked, since many years have passed since the cancer. Also surprised, since I haven’t even been in contact with an oncologist and felt “unwanted” by the health care. | Positive but surprised |  |
| Fantastic! I felt a huge reverence over this offer, considering it was a long time ago since I got my cancer diagnosis and went through all the treatments – THANK YOU! | Fantastic |  |
| Scary, but still positive. | Scary but positive | Emotional but |
| At first, I was afraid, didn’t want to “wake” everything to life again. But, after we talked about it at home, it was a given. | Afraid but positive | positive responses |
| I was afraid that I would have the genes that enhances the risk for ovarian cancer. When I was offered the truth, I thought that I had to take it and to have the ovaries removed if I get the offer. | Afraid but positive |  |
| I want to see if it’s hereditary. | Wanting knowledge | Not responses to the |
| An interest in knowing if the breast cancer I had is hereditary. | Wanting knowledge | question but positive |
| *What was the main reason for choosing to participate in the study?* |  |  |
| I have a daughter who is almost 17 years old, and I have obviously, over the years, pondered the heredity. | Daughter | Family’s risk |
| I have a daughter (who was 9 months old when I realized that I had a lump in my breast), whom I obviously have been worried for, considering the heredity of breast cancer. | Daughter |  |
| Because I have a daughter. | Daughter |  |
| Because I have a 12-year-old daughter. | Daughter |  |
| Because of my daughter and my sister’s daughters. | Daughters |  |
| I wanted to get an answer on if I carried the gene, for descendants. | Descendants |  |
| Because my son has gotten Burkitt’s lymphoma and I wanted to know if it could be because of my breast cancer. | Son |  |
| I see a need for the information, a piece of the puzzle for enhanced care of myself and a guidance about how I want my daughter to be treated. | Myself and daughter | Own and family’s risk |
| Have always wanted to know if I carry a genetic breast cancer, considering my children, and for my own sake. | Children and own sake |  |
| A safety, both for me and my family, to know. | Myself and family |  |
| Of course, you want to know if you have an enhanced risk for additional cancer diagnoses, or if my children have. | Own risk and children’s risk |  |
| I wanted to know the result to be able to inform my family and relatives. And, for my own sake, the opportunity of getting prophylactic treatment. | Family and own prophylactic treatment |  |
| Foremost, to know if I should do any preventive efforts to avoid being affected again. Operations on the other breast or uterus. | Own prevention | Own risk |
| I have not felt prioritized by the healthcare and have experienced that it has been difficult to be taken seriously. For instance, to get additional mammography (now I only get screening according to the screening program of the “healthy” breast). | Own prevention |  |
| The reason why I got breast cancer. | Reason why | Increased knowledge |
| Knowledge is always positive. | Knowledge positive |  |
| Good to know, don’t have to worry about there being a genetic component. | Knowledge good |  |
| In consultation with my son and husband, we decided that we wanted to know. | Knowledge |  |
| Get an answer! | Get answer |  |
| I’m adopted. Didn’t know if I had a hereditary alteration in the genes. | Adopted |  |
| It felt current, since I was “young” when I got the cancer. | Felt current |  |
| To support medical research. I’m going through investigation due to metastasis in the lung, so I’m already enrolled within the healthcare system. | Medical research | Support research |
| Everything I can do to lead research forward, I obviously want to take part in. | Research |  |
| *What was your experience of being informed of the result from the genetic analysis through a letter?* |  |  |
| Considering that it was good information for us, it felt quite enough. | Quite enough | Positive responses |
| Good. For a neutral result, a letter is best. | Good |  |
| It was good. | Good |  |
| Good! | Good |  |
| Good. Got the information before, on how I was going to be contacted if I did/if I didn’t carry the gene. | Good |  |
| I felt safe. | Safe |  |
| Positive. I don’t have any problems with getting such things through letters. | Positive |  |
| Yes, exciting. | Exciting |  |
| Feels OK when the information is positive. | OK |  |
| Since I got positive information, it felt totally OK. | Totally OK |  |
| Felt totally OK. | Totally OK |  |
| Totally OK. | Totally OK |  |
| I have always thought that my cancer was hereditary. Totally OK to get the answer through a letter. | Totally OK |  |
| Totally OK, was prepared for that the answer would take longer. | Totally OK |  |
| Totally OK, but easier when the answer was that I didn’t demonstrate heredity. | Totally OK |  |
| Totally OK! I just wanted to know. | Totally OK |  |
| It feels soothing. | Soothing |  |
| A clear answer, which is very important, so that there aren’t any question marks when you have read the answer. | Clear answer |  |
| A relieve that it wasn’t genetic. | Relief |  |
| Since all communication was through letters, it felt natural to continue through a letter. | Natural continuing |  |
| Totally OK, but would have liked to have the opportunity to ask questions through a dialog. | Totally OK but more dialog | Additional oral conversation |
| OK, since it was a positive answer. But, a conversation is preferred. | OK but conversation |  |
| It obviously felt a bit jittery and worrisome, but at the same time it was nice that the information for my part was calming. | Feeling worried but  information calming | Emotional but positive response |
|  |  |  |
| **Women with a pathogenic variant, *n*=4** |  |  |
| *What was your experience of being offered genetic testing through a letter?* |  |  |
| I was happy. | Happy | Positive responses |
| I was happy that I got the opportunity to see if it was genetic. | Happy |  |
| Positive. | Positive |  |
| Sad to tear up old worries, but grateful that it’s checked out, not least for my daughter’s sake. | Sad but grateful | Emotional but positive response |
| *What was the main reason for choosing to participate in the study?* |  |  |
| To know if you have the gene could protect sisters and daughter in the future. | Sisters and daughters | Family’s risk |
| Because I have a daughter today. | Daughter |  |
| To get knowledge and be able to prevent the preventable, and inform the children so that they are attentive to symptoms. | Prevention and children | Own and family’s risk |
| I want to prevent the risk for additional breast cancer and ovarian cancer. | Prevention | Own risk |
| *What was your experience of being informed of the result from the genetic analysis through a telephone call and subsequent genetic counseling?* |  |  |
| Good information.  It was good. | Good  Good | Positive responses |
| More than a week between the first conversation with the nurse and the conversation with the physician. Had time to think and worry a little during this time. Otherwise, totally OK. | Feeling worried  but totally OK | Emotional but positive response |
| I was a bit sad. | Sad | Emotional response |
